# Cellular Abnormalities Induced by High Glucose in Mixed Glial Cultures Are Maintained, Although Glucose Returns to Normal Levels

**DOI:** 10.3390/brainsci15090952

**Published:** 2025-09-01

**Authors:** Brandon Isai Herrera Solis, Frida Guerrero-Padilla, Elvia Mera Jiménez, Juan Manuel Vega López, María de Jesús Perea-Flores, Octavio Rodríguez-Cortés, Martha Edith Macías Pérez, Maricarmen Hernández-Rodríguez

**Affiliations:** 1 Laboratorio de Cultivo Celular, Neurofarmacología y Conducta, Sección de Estudios de Posgrado e Investigación, Escuela Superior de Medicina, Instituto Politécnico Nacional, Ciudad de México 11340, Mexico; brandon2700oct@gmail.com (B.I.H.S.); fridaguep@gmail.com (F.G.-P.); elviamj@gmail.com (E.M.J.); marthita_e23@yahoo.com.mx (M.E.M.P.); 2 Escuela Nacional de Ciencias Biológicas, Instituto Politécnico Nacional, Ciudad de México 11340, Mexico; vegaj5648@gmail.com; 3 Centro de Nanociencias Micro y Nanotecnologías, Instituto Politécnico Nacional, Ciudad de México 07700, Mexico; mpereaf@ipn.mx; 4 Laboratorio de Inflamación y Obesidad, Sección de Estudios de Posgrado e Investigación, Escuela Superior de Medicina, Instituto Politécnico Nacional, Ciudad de México 11340, Mexico; octaviordzc@yahoo.com.mx

**Keywords:** metabolic memory, high glucose, mixed glial cell culture

## Abstract

**Background:** Metabolic memory refers to the long-term adverse effects of short-term disturbances in glucose metabolism. Recent evidence indicates that hyperglycemia-induced metabolic memory contributes to sustained cellular damage even after glycemic control, driven by increased production of reactive oxygen species (ROS), activation of inflammatory pathways, and accumulation of advanced glycation end products (AGEs). Although well characterized in endothelial and smooth muscle cells, this phenomenon may also occur in other cell types, including glial cells. **Objective:** This study aimed to evaluate the persistence of high-glucose (HG)-induced alterations after returning to normal glucose (NG) conditions in primary mixed glial cell (MGC) cultures. **Methods:** Primary MGCs were obtained from neonatal Wistar rat pups and cultured under three conditions for 21 days: NG (5.5 mM glucose), HG (25 mM glucose), and HG-NG (14 days in HG followed by 7 days in NG). Cell proliferation, apoptosis, ROS production, lipid peroxidation, mitochondrial activity, TNF-α, IL-6, and AGE formation were assessed. **Results:** MGCs cultured under HG and HG-NG conditions exhibited reduced proliferation without increased apoptosis. Both HG and HG-NG conditions promoted ROS overproduction accompanied by reduced mitochondrial activity, whereas only HG increased lipid peroxidation. Notably, TNF-α and AGE levels were elevated in both HG and HG-NG conditions, while IL-6 production decreased exclusively in HG-NG. **Conclusions:** These findings demonstrate the persistence of deleterious effects induced by HG in MGCs, even after restoration to NG conditions.

## 1. Introduction

Diabetes is a systemic metabolic disorder characterized by elevated blood glucose levels, known as hyperglycemia, resulting from defects in insulin secretion, insulin action, or both [1]. Diabetes leads to complications that are typically classified into two categories: microvascular complications, including retinopathy, nephropathy, and neuropathy, and macrovascular complications, such as ischemic heart disease, peripheral vascular disease, and cerebrovascular disease [1]. Although the detrimental effects of diabetes on the brain have often been underestimated, growing evidence shows that individuals with diabetes exhibit cognitive decline and structural alterations in the brain [2]. Furthermore, diabetes has been increasingly implicated in the pathogenesis of neurodegenerative disorders, including Alzheimer’s disease (AD) and Parkinson’s disease (PD) [3].

High glucose (HG) levels induce a wide range of cellular disturbances in both glial cells and neurons [4]. In diabetic patients, chronic hyperglycemia promotes pathological changes in the brain by exposing astrocytes and microglia to sustained glucose elevation, leading to increased reactive oxygen species (ROS) production and a persistent pro-inflammatory state [5]. Astrocytes and microglia play a central role in maintaining brain homeostasis: at the macro level, they regulate nervous system architecture, cell number, and circuit organization, while at the molecular level, they sustain ion, neurotransmitter, and metabolite balance [6].

The impact of hyperglycemia on astrocytes and microglia has been extensively studied in isolation, using enriched primary cultures or established cell lines. For instance, Li et al. (2018) reported that primary astrocytes cultured under HG conditions exhibited significantly slower proliferation than those cultured under normal glucose (NG) conditions, due to cell cycle arrest in the G2/M phase without increased apoptosis [7]. Similarly, human astrocytic cells exposed to HG showed elevated ROS and tumor necrosis factor-α (TNF-α) production [8]. In microglia, HG conditions enhanced oxidative and inflammatory responses in the BV-2 cell line [9]. Moreover, human microglia cultured in HG for 12 days exhibited early differentiation into an M2b-like phenotype, followed by a shift toward an M1-like phenotype, suggesting that chronic hyperglycemia drives gradual microglial polarization toward an M2-like state characterized by pro-inflammatory cytokine release [10].

Studying mixed glial cell (MGC) cultures, which contain both astrocytes and microglia, is particularly relevant because they allow for an investigation of the dynamic interactions and cross-talk between these cell types in the central nervous system (CNS) [6]. Disruption of this communication may contribute to the development of neurodegenerative disorders [6].

Furthermore, recent studies have begun to explore the dynamic interactions between astrocytes and microglia in MGC cultures. It has been reported that isolated primary microglial cells exhibited an activated amoeboid morphology, whereas microglia within MGCs displayed a ramified morphology similar to that observed in the brains of wild-type mice. This finding suggests that mixed glial cultures more closely reproduce the in vivo microglial phenotype [11], underscoring the importance of studying glial communication to better understand pathological conditions affecting the brain, including diabetes.

In addition, it has been demonstrated that the detrimental effects of HG exposure can persist even after returning to NG conditions. This phenomenon, known as “metabolic memory” [12], helps explain how irreversible alterations in cellular function caused by hyperglycemia may significantly contribute to the development and progression of diabetes and its long-term complications [13]. Notably, most current evidence on metabolic memory derives from studies in endothelial and smooth muscle cells, where its mechanisms include inflammation, oxidative stress, accumulation of advanced glycation end products (AGEs), and epigenetic modifications—processes that are interconnected [14]. Oxidative stress is considered a major driver of metabolic memory. For example, Ihnat et al. (2007) showed that human umbilical vein endothelial cells and retinal cells exposed to HG for two weeks, followed by one week under NG, continued to express HG-induced oxidative stress markers [15]. Another mechanism involves AGE formation, as AGEs can bind to proteins and alter their structure and function, or trigger cellular dysfunction through interaction with their receptors, thereby activating intracellular pathways that promote inflammation and oxidative stress [16].

However, the metabolic memory phenomenon has not yet been described in glial cells. Therefore, the present study aimed to determine whether the deleterious effects induced by HG in MGC cultures persist after restoration to NG conditions.

## 2. Materials and Methods

### 2.1. Mixed Glial Cell (MGC) Culture

All animal procedures were conducted in accordance with the Mexican Official Standard NOM-062-ZOO-1999 and the Biosecurity Committee of CBS-ESM-IPN (protocol ESM-CBS-03/8-10-2018). MGC cultures were prepared as previously described [17]. Briefly, neonatal Wistar rat pups of both sexes (P1–P5) were rinsed with 70% ethanol, decapitated with sterile sharp scissors, and the heads were immediately immersed in 70% ethanol. This procedure was performed for a total of five rat pups, after which the heads were transferred to a saline solution. The brains were dissected, meninges removed, and cortices isolated and placed in a Petri dish with cold DMEM (5.5 mM glucose; 11885084, GIBCO) on ice.

The tissue was mechanically dissociated by pipetting up and down 10 times with a sterile 10 mL pipette. The resulting cell suspension was passed through a 100 μm cell strainer and centrifuged at 2000 rpm for 10 min. The cell pellet was resuspended in DMEM (5.5 mM glucose) supplemented with 10% fetal bovine serum (FBS), 120 U/mL penicillin, and 12 mg/10 mL streptomycin, and then seeded into five culture flasks (75 cm^2^; approximately one brain per flask). Cells were maintained in an incubator (Nuaire, In-VitroCell NU-8600, South Wales, UK) at 36.5 °C in a humidified atmosphere with 5% CO_2_ for 5 days. Medium was subsequently replaced every 3 days until confluence was reached.

Because primary MGC cultures consist mainly of astrocytes (~80%) and microglia (~20%) [18], phenotypic characterization of both cell types was performed by immunohistochemistry. Astrocytes were identified by glial fibrillary acidic protein (GFAP) immunostaining (Santa Cruz Biotechnology, sc-33673; Dallas, TX, USA) with Alexa Fluor 488 (green), while microglia were identified by ionized calcium-binding adapter molecule 1 (Iba1) immunostaining (Santa Cruz Biotechnology, sc-32725; Dallas, TX, USA) with Alexa Fluor 594 (red). The proportion of astrocytes and microglia was determined from images of five randomly selected fields (20×).

### 2.2. Glucose Conditions

**Figure 1:**
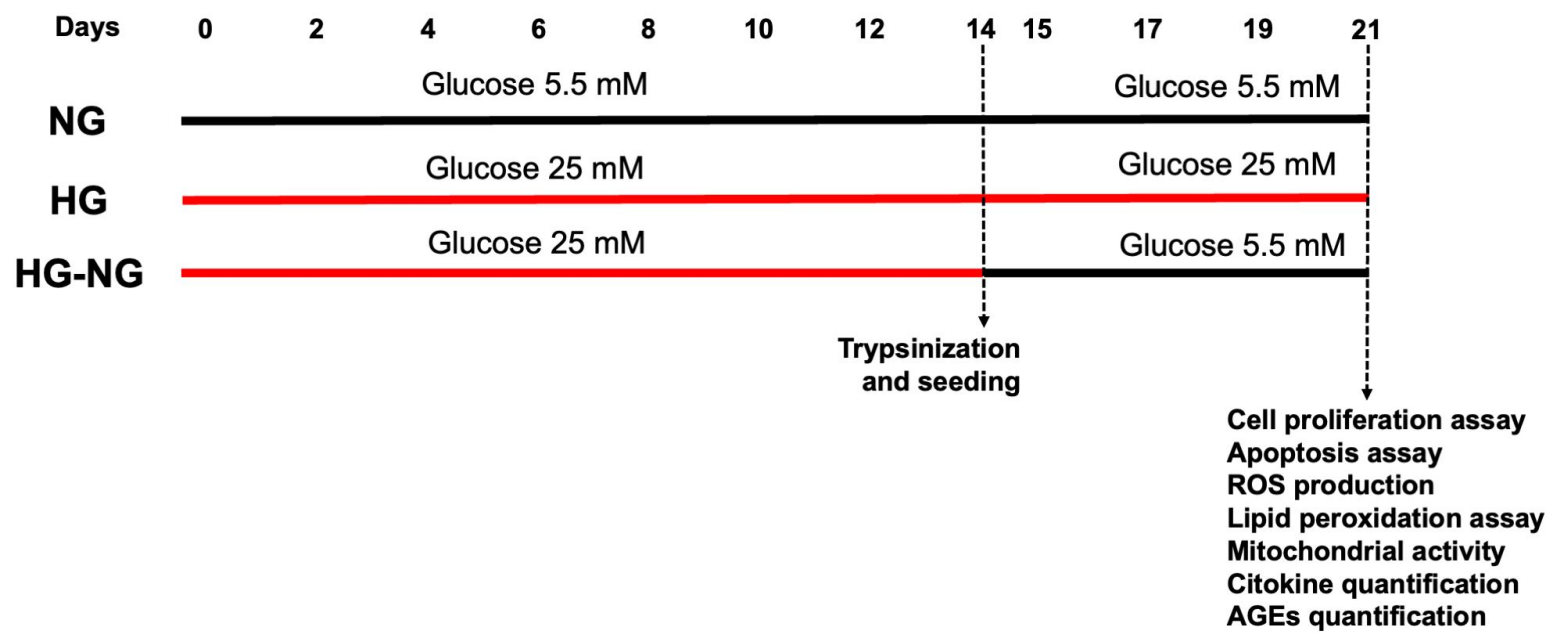
General scheme of glucose conditions and assays performed on MGC cultures.

MGCs were cultured under three different glucose conditions, as shown in Figure 1: (i) NG condition, cells maintained in 5.5 mM glucose for 21 days; (ii) HG condition, cells maintained in 25 mM glucose for 21 days; and (iii) HG-NG condition, cells maintained in 25 mM glucose for 14 days followed by 5.5 mM glucose for 7 days. Cultures were kept in flasks until day 14 in an incubator (Nuaire In-VitroCell NU-8600, UK) at 36.5 °C in a humidified atmosphere with 5% CO_2_. Medium was replaced every 3 days. At day 14, cells were trypsinized and seeded into 24- or 96-well plates for subsequent assays, as previously described [19].

Glucose concentrations were selected based on the study by Li et al. (2018), which first reported the irreversible effects of HG on the reduced proliferation rate of astrocytes [7]. The duration of exposure was determined in accordance with previous studies investigating the development of metabolic memory in human umbilical endothelial cells and ARPE-19 retinal cells [15,20].

### 2.3. Cell Proliferation Assay

Cell proliferation was assessed using the sulforhodamine B (SRB) assay, which measures cellular protein content as an index of cell density [21]. Briefly, after 14 days of exposure to the different glucose conditions, cells from the NG, HG, and HG-NG groups were trypsinized, and 25,000 cells in 100 μL were seeded into 96-well culture plates and maintained under the same culture conditions. On day 21, cells were fixed in situ by adding 50 μL of cold 20% (*w*/*v*) trichloroacetic acid (TCA) directly to the culture medium, followed by incubation for 60 min at 4 °C. The supernatant was then discarded, and plates were washed four times with tap water and air-dried.

TCA-fixed cultures were stained for 30 min at room temperature with 100 μL of 0.057% SRB solution prepared in 1% acetic acid. Excess dye was removed by washing the plates four times with 1% acetic acid. Protein-bound dye was solubilized by adding 100 μL of 10 mM Tris base (pH 10.5), and the plates were incubated for 5 min at room temperature on an orbital shaker (100 rpm). Optical density (OD) was measured at 540 nm using a microplate reader (Thermo Scientific™ Fluoroskan™, Waltham, MA, USA).

For data analysis, the OD of MGCs cultured under NG conditions was set as the baseline (100%). OD values of MGCs cultured under HG and HG-NG conditions were expressed relative to this baseline, representing the percentage change in cell density compared with NG. Data were obtained from three independent experiments and are expressed as mean ± standard error of the mean (SEM).

### 2.4. Apoptosis Assay

Apoptotic cells were quantified by Annexin V/7-AAD staining followed by flow cytometry, a method widely applied to eukaryotic cells, including astrocytes [22]. Dual staining with Annexin V and 7-AAD allows discrimination of viable cells (Annexin V^−^/7-AAD^−^), apoptotic cells (Annexin V^+^), and necrotic cells (Annexin V^−^/7-AAD^+^).

Briefly, after 14 days of exposure to glucose conditions, cells from NG, HG, and HG-NG groups were trypsinized, and 1 × 10^6^ cells/mL were seeded into 24-well microplates and cultured until day 21. Cells were then harvested, washed with PBS, and resuspended in 200 μL of binding buffer (4 mM KCl, 0.75 mM MgCl_2_, 85 mM CaCl_2_, 140 mM NaCl, and 10 mM HEPES in distilled water). The suspension was incubated with 2.5 μL Annexin V (BioLegend, 640945; San Diego, CA, USA) and 1 μL 7-AAD (BioLegend, 420404; San Diego, CA, USA) for 15 min at room temperature (25 °C) in the dark.

To establish cutoff points for apoptosis and necrosis, autofluorescence and compensation controls were included. Unstained cells were acquired to determine autofluorescence in the FL-1 (Annexin V) and FL-3 (7-AAD) channels. Compensation values were then set using single-stained samples to correct for spectral overlap. Experimental samples were subsequently acquired, with 10,000 events recorded per sample on a FACSCanto flow cytometer (BD Biosciences, CA, USA). Data were analyzed with BD FACSDiva 9.0 software(BD Biosciences, CA, USA).

### 2.5. ROS Production

Intracellular ROS levels were assessed using 2′,7′-dichlorofluorescin diacetate (DCFH-DA). After 14 days of culture under different glucose conditions, cells from the NG, HG, and HG-NG groups were trypsinized and seeded onto 24-well plates containing circular coverslips at a density of 1 × 10^5^ cells/well. Cultures were maintained until day 21 under the same conditions described above.

At the endpoint, cells were washed twice with 500 μL PBS (10 mM, pH 7.4) and incubated with 200 μL of 10 μM DCFH-DA in PBS for 30 min at room temperature in the dark. Cells were then washed twice with PBS, fixed with 200 μL of 4% paraformaldehyde in PBS for 10 min at room temperature, and rinsed again with PBS. Coverslips were mounted on slides, and images from five randomly selected fields per coverslip were acquired using a multiphoton-confocal microscope (LSM 710 NLO, Carl Zeiss, Oberkochen, Germany) at Ex/Em 493/540 nm (laser excitation: 488 nm; 2% transmittance).

Fluorescence intensity was quantified with ImageJ software Version 1.53T. Cytoplasmic regions were manually outlined, and the mean gray value of each cell was measured. ROS production was normalized to cell mass determined by SRB assay across pooled replicates. For normalization, the mean gray value of NG cells was set to 1, and values from HG and HG-NG groups were expressed relative to this baseline. Data represent the mean ± SEM of three independent experiments [23].

### 2.6. Lipid Peroxidation Assay

Lipid peroxidation was assessed using a fluorometric quantification kit (Sigma-Aldrich, Saint Louis, MO, USA, #MAK568), which detects oxidized lipids with excitation/emission wavelengths of 496/519 nm. The assay primarily measures malondialdehyde (MDA), a well-established marker of lipid peroxidation and oxidative stress.

At day 14 of culture, MGC from NG, HG, and HG-NG groups were trypsinized and seeded onto 24-well plates containing circular coverslips at a density of 1 × 10^5^ cells/well, and incubated under the same conditions until day 21. At the endpoint, 50 μL of the master reaction mix was added to each well and incubated for 60 min at room temperature in the dark. Cells were then washed three times with PBS, and coverslips were mounted on slides.

Fluorescence images were acquired from five randomly selected fields per coverslip using a fluorescence microscope (Axio Scope A1, Carl Zeiss, Oberkochen, Germany) equipped with a filter cube (excitation 485/17 nm; emission 560/18 nm). Fluorescence intensity in the green channel was quantified with ImageJ software Version 1.53T [24]. Values were normalized to cell mass determined by SRB assay across pooled replicates. Data are presented as mean ± SEM from three independent experiments.

### 2.7. Mitochondrial Activity

Mitochondrial activity was assessed using the MTT assay, which measures the conversion of the yellow dye MTT [3-(4,5-dimethylthiazol-2-yl)-2,5-diphenyltetrazolium bromide] into an insoluble purple formazan product via mitochondrial reductases [25]. This assay is highly sensitive, allowing detection of small changes in mitochondrial function and cellular stress induced by deleterious agents [26].

At day 14 of culture, MGCs from NG, HG, and HG-NG groups were trypsinized and seeded at 25,000 cells/100 μL per well in 96-well plates, maintained under the previously described conditions. On day 21, 20 μL of MTT solution (5 mg/mL in PBS) was added to each well and incubated for 4 h at 37 °C in a CO_2_ incubator, protected from light under a layer of mineral oil. A negative control was prepared by adding 20 μL of MTT stock solution to 100 μL of medium without cells.

After incubation, the culture medium was carefully removed, and 150 μL of 4 mM HCl in isopropanol was added to solubilize formazan crystals. OD was measured at 590 nm using a microplate reader (Thermo Scientific™ Fluoroskan™, MA, USA). Mitochondrial activity was normalized to cell mass determined by SRB assay. OD values from negative control wells were subtracted, and the OD of NG cells was set as 100%. OD values of HG and HG-NG groups were expressed relative to this baseline. Data represent the mean ± SEM of three independent experiments.

### 2.8. Citokine Quantification

At day 14 of culture, MGCs from NG, HG, and HG-NG groups were trypsinized and seeded into 24-well plates at a density of 1 × 10^5^ cells/well, and cultured until day 21 under the previously described conditions. At this time, supernatants were collected, with 50 μL used for TNF-α measurement and 100 μL for IL-6 measurement, each performed in triplicate.

TNF-α concentration was determined by flow cytometry using a FACSCanto II (BD Biosciences, San Jose, CA, USA) and the BD Cytometric Bead Array Rat kit (CBA, BD Biosciences, 558309; assay range: 40–10,000 pg/mL), according to the manufacturer’s instructions. Data were analyzed using FCAP Array software v. 3.0 (BD Biosciences) and expressed as pg/mL based on standard curves.

IL-6 levels were quantified using a Rat IL-6 ELISA kit (Sigma-Aldrich, RAB0311-1KIT; assay range: 30–10,000 pg/mL) following the manufacturer’s instructions. Absorbance was read with a microplate reader (Thermo Scientific™ Fluoroskan™, Waltham, MA, USA), and results were expressed as pg/mL based on standard curves.

Cytokine concentrations were normalized to cell mass determined by SRB assay across pooled replicates. Data represent the mean ± SEM of three independent experiments.

### 2.9. AGEs Quantification

At day 14 of culture, MGCs from NG, HG, and HG-NG groups were trypsinized and seeded into 24-well plates at a density of 1 × 10^5^ cells/well, and cultured until day 21 under the previously described conditions. At the endpoint, supernatants were discarded, and cells were lysed with 200 μL of Radioimmunoprecipitation (RIPA) buffer on ice for 5 min. Lysates were collected into 1.5 mL tubes and centrifuged at 21,130× *g* for 10 min.

AGE protein levels were measured by competitive Enzyme-Linked Immunosorbent (ELISA) using 100 μL of undiluted cell lysate and an AGE assay kit (Abcam, ab238539; assay range: 0.36–100 µg/mL). Optical density was read at 450 nm using a microplate reader (Thermo Scientific™ Fluoroskan™, MA, USA). Results were calculated based on a standard curve generated with AGE-BSA and expressed as µg/mL AGE-BSA equivalents.

AGE values were normalized to cell mass determined by SRB assay across pooled replicates. Data represent the mean ± SEM of three independent experiments.

### 2.10. Statistical Analysis

Data were analyzed using GraphPad Prism software Version 10 (GraphPad, San Diego, CA, USA) and are presented as mean ± SEM from three independent experiments (*n* = 3–8 biological replicates per group). Normality of each dataset was assessed using the Shapiro–Wilk test (α = 0.05). Statistical comparisons among groups were performed by one-way analysis of variance (ANOVA) followed by Tukey’s post hoc test. Differences were considered statistically significant at *p* < 0.05.

## 3. Results

### 3.1. MGC Characterization

**Figure 2:**
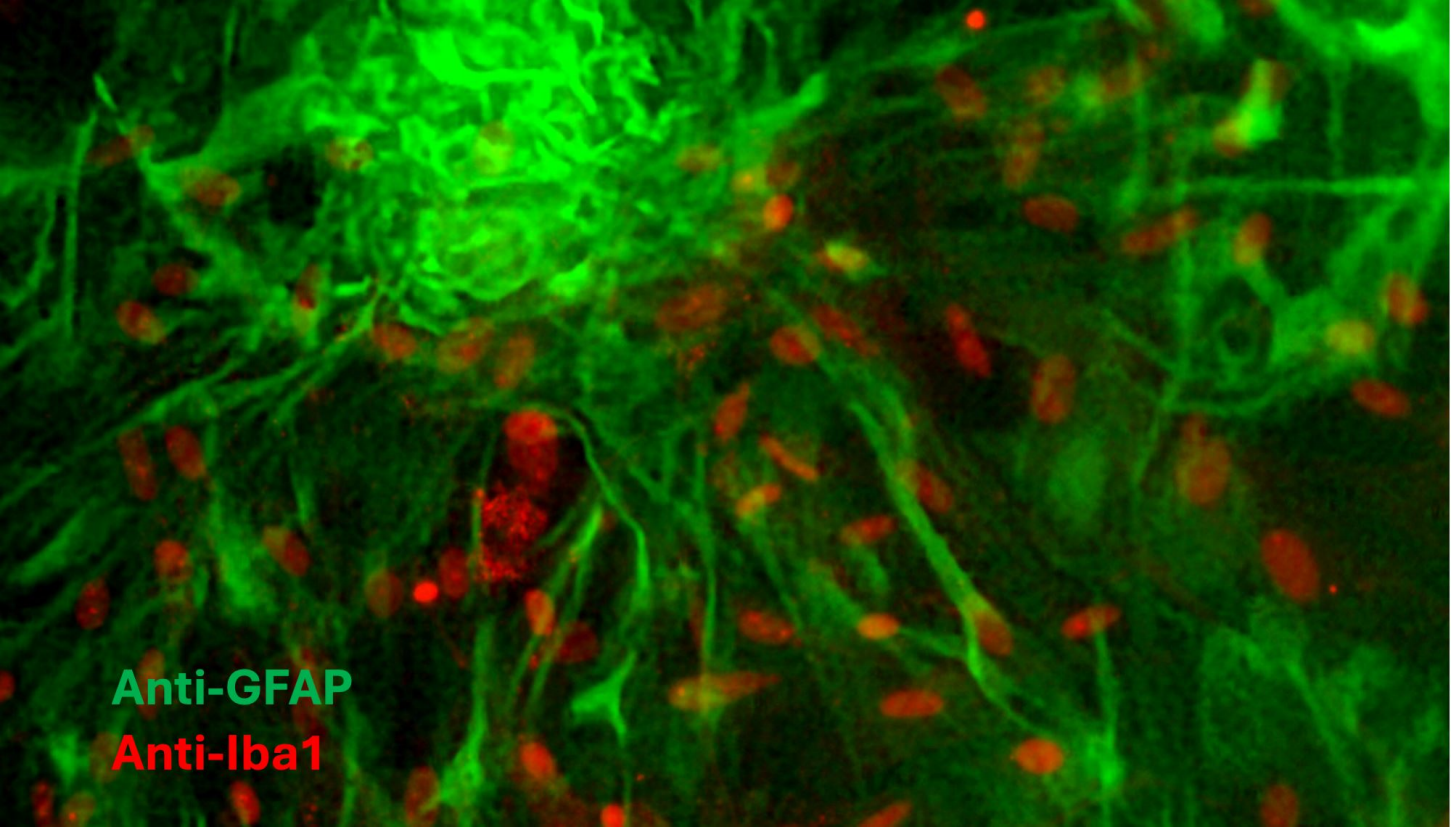
Representative image of MGC culture characterization. Astrocytes were identified by anti-GFAP labeling (green), and microglia were labeled with anti-Iba1 (red). Images were acquired using a fluorescence microscope (Axio Scope A1, Carl Zeiss, Oberkochen, Germany) at 20× field.

To assess the effects of glucose treatments on MGC, immunohistochemical characterization of astrocytes and microglia derived from hemispheres of neonatal Wistar rat pups was performed (Figure 2). The proportion of astrocytes and microglia in the culture was consistent with previous reports, accounting for 77.4% and 22.6%, respectively [18].

### 3.2. HG Produces an Irreversible Decrease in Cell Proliferation in MGC

**Figure 3:**
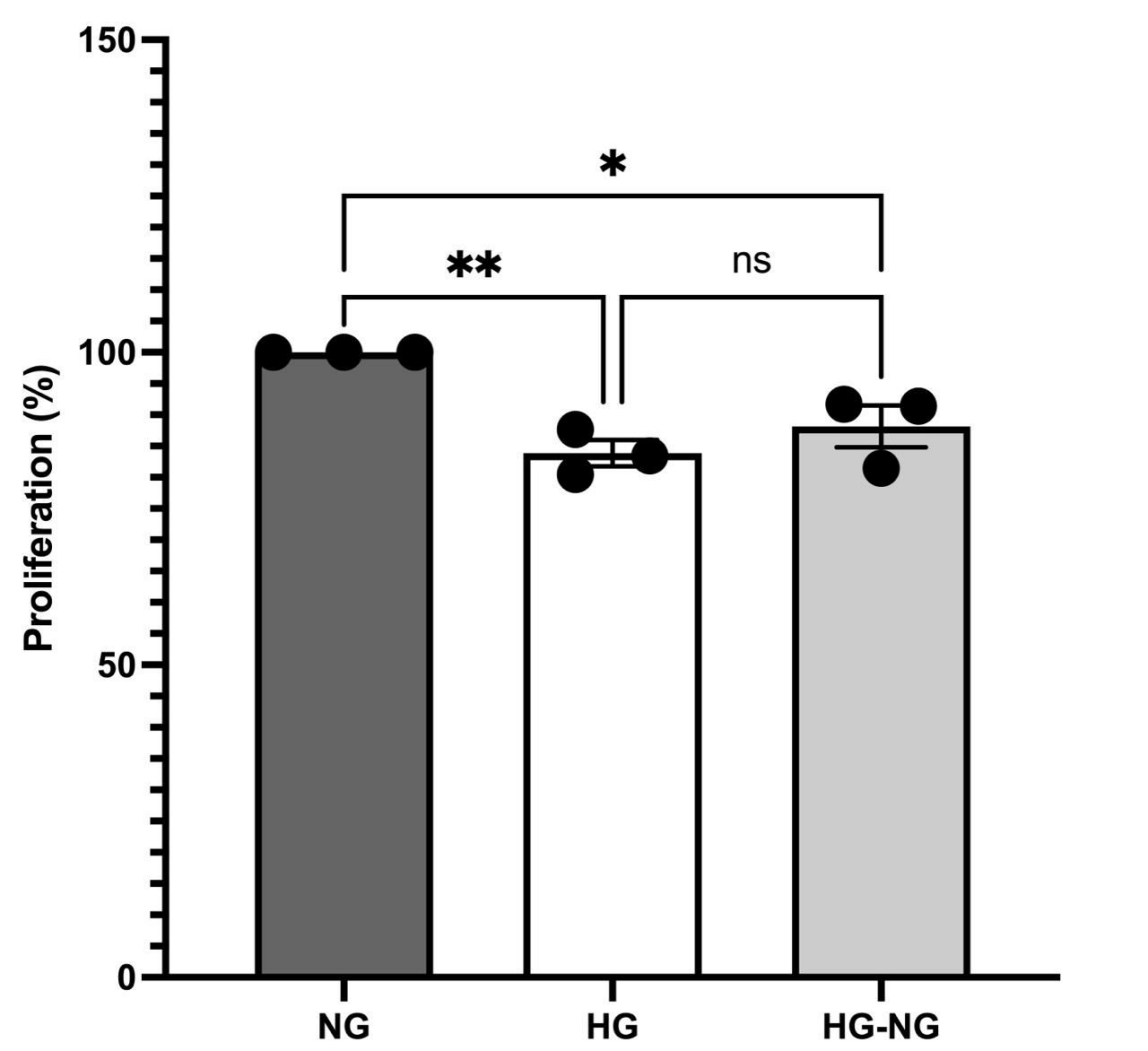
Changes in cell proliferation in MGC cultures under different glucose conditions were assessed using the SRB assay. Data are from three separate experiments and represent the mean ± SEM. Statistical analyses were performed by one-way ANOVA; * *p* < 0.05 and ** *p* < 0.01. For between-group comparisons, Tukey’s test was used as a post hoc test.

Cell proliferation was assessed on day 21 of culture under different glucose conditions (Figure 3). At day 14, MGC from all three groups were seeded at a density of 5 × 10^4^ cells/well. No significant differences in proliferation were observed on day 15.

By day 21, MGC cultured under HG conditions exhibited a 16.1 ± 2.1% decrease in proliferation compared to NG cells (*p* = 0.0060). Restoration of NG from day 15 in the HG-NG group did not rescue proliferation, resulting in an 11.82 ± 3.36% decrease relative to NG (*p* = 0.0248). No significant difference was observed between HG and HG-NG groups (*p* = 0.4306), indicating that HG induces a persistent reduction in MGC proliferation.

**Figure 4:**
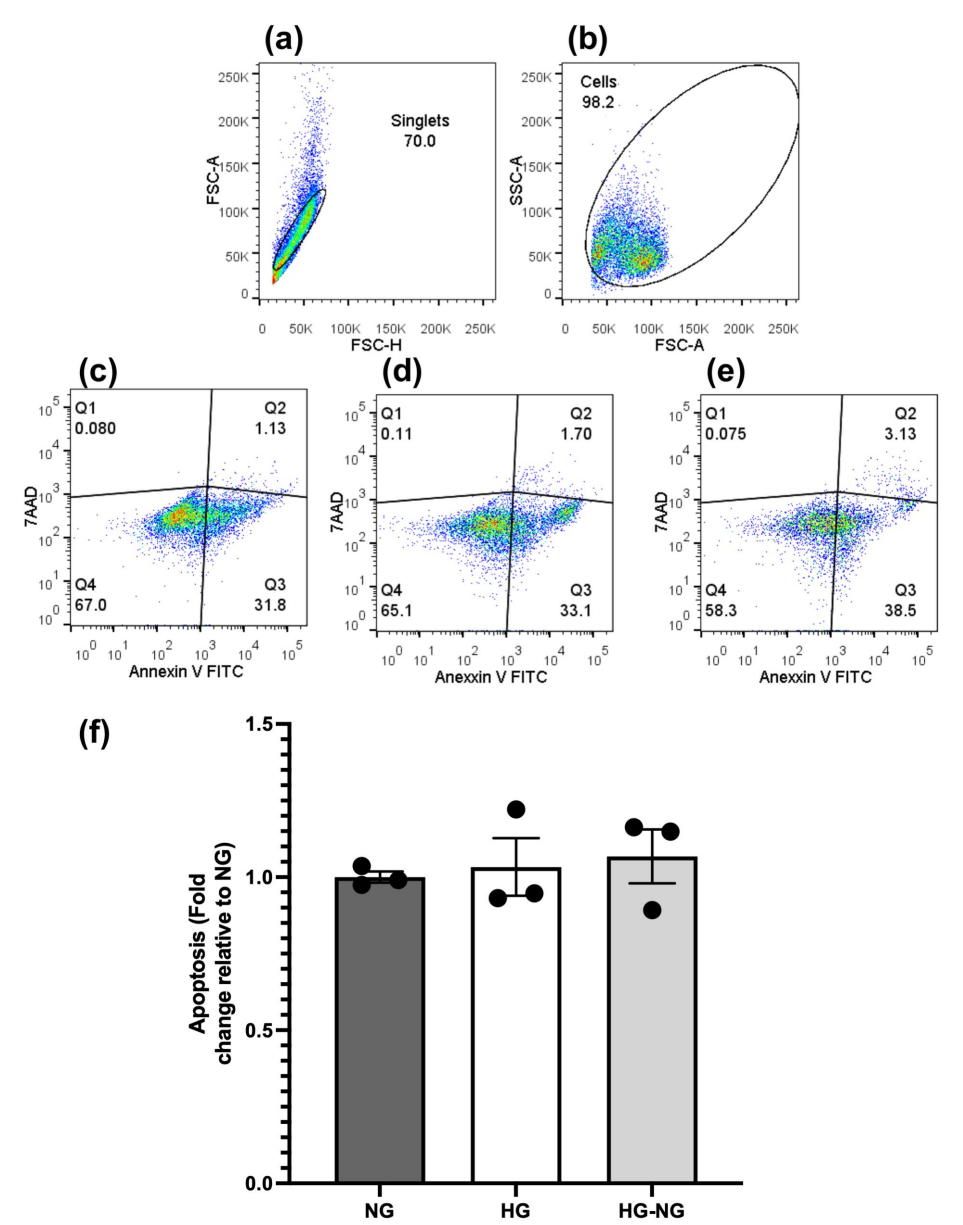
Changes in apoptosis in MGC cultures under different glucose conditions were determined by double staining with Annexin V and 7AAD. The analysis strategy was as follows: (**a**) Singlet events (to discard grouped cells); (**b**) Size (FSC) vs. Granularity (SSC) dot plot. Percentage of apoptosis in (**c**) NG, (**d**) HG, and (**e**) HG-NG groups. In all cases, the percentage of total apoptosis is the sum of the events in the quadrants Q3 (early apoptosis; Annexin V^+^/7-AAD^−^) + Q4 (late apoptosis; Annexin V^+^/7-AAD^−^). (**f**) Comparison of the percentage of apoptosis between the groups. A total of 5000 events were analyzed for each group. Data are from three separate experiments and represent the mean ± SEM. Statistical analyses were performed by one-way ANOVA. For between-group comparisons, Tukey’s test was used as a post hoc test.

To determine whether the reduced proliferation observed in the HG group was due to apoptosis, MGC were subjected to double staining with Annexin V/7-AAD on day 21. The analysis strategy included gating for singlet events to exclude cell aggregates (Figure 4a) and assessing cell size (FSC) versus granularity (SSC) (Figure 4b). Representative apoptosis data for NG, HG, and HG-NG groups are shown in Figure 4c–e, respectively.

Quantitative analysis (Figure 4f) revealed no significant differences in apoptosis between HG and HG-NG groups compared to NG (*p* = 0.95 and *p* = 0.8072, respectively). Furthermore, no differences in proliferation were observed between HG and HG-NG groups (*p* = 0.9428), indicating that the HG-induced reduction in MGC proliferation is not mediated by apoptosis.

### 3.3. HG Produces an Irreversible Increase in the Production of ROS but Not in Lipid Peroxidation

**Figure 5:**
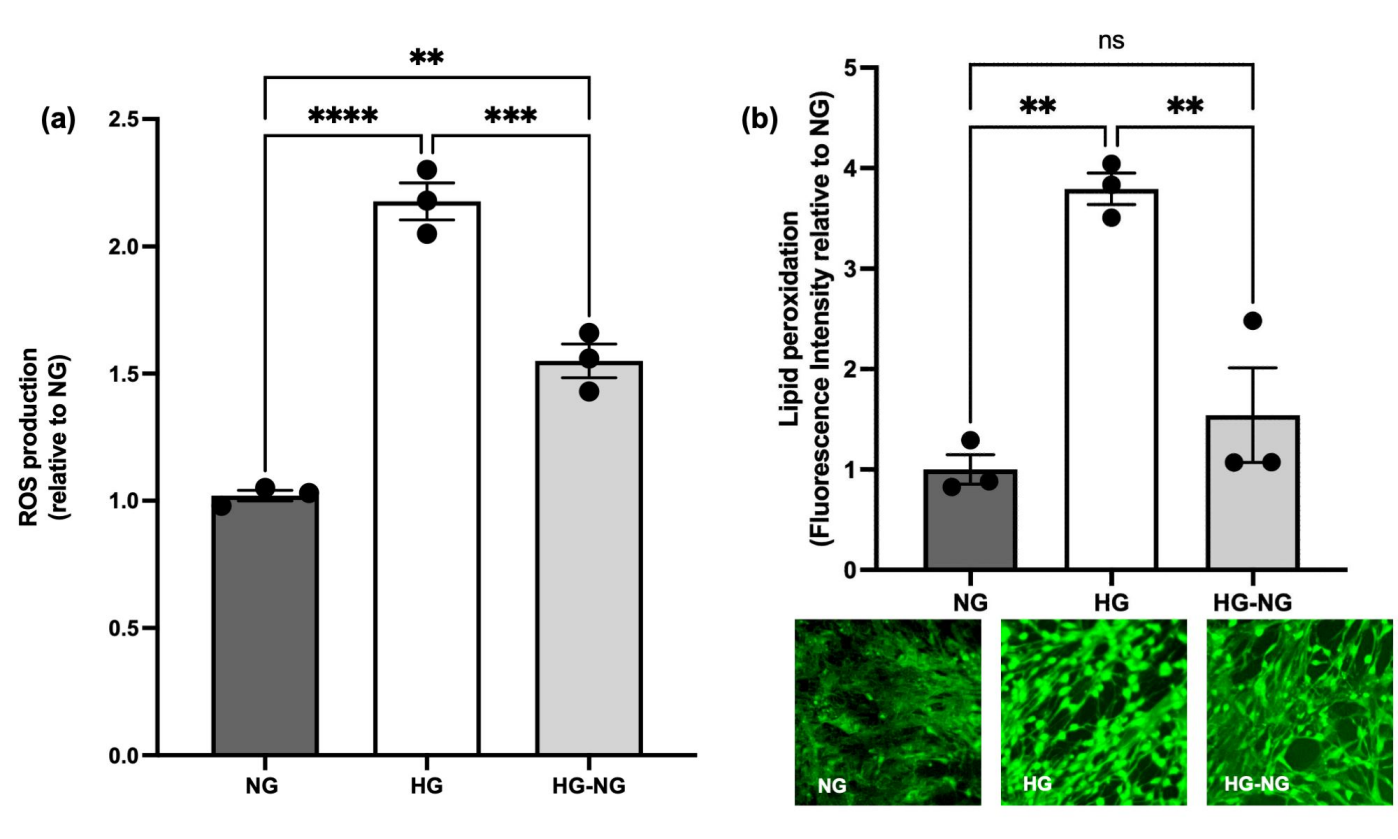
ROS production and lipid peroxidation levels of MGC cultured under different glucose conditions. (**a**) ROS production evidenced by DCFH-DA staining. Images were obtained by Multiphoton-Confocal Microscope (LSM 710 NLO, Carl Zeiss, Germany) at Ex/Em 493/540 nm; laser excitation 488; 2% transmittance. Average fluorescence intensity was calculated using ImageJ software. (**b**) Lipid peroxidation in MGC incubated under different glucose conditions, evidenced by MDA fluorometric dying; representative images are shown at the bottom. Images were obtained by Fluorescence Microscope (Axio Scope A1, Carl Zeiss Germany) employing a filter cube set 40 (Carl Zeiss Germany). Average fluorescence intensity was calculated for MGC incubated under different glucose conditions using ImageJ software; representative photographs are shown. Data are from three separate experiments and represent the mean ± SEM. Statistical analyses were performed by one-way ANOVA; ** *p* < 0.01, *** *p* < 0.001, and **** *p* < 0.0001. For between-group comparisons, Tukey’s test was used as a post hoc test.

As shown in Figure 5a, MGC cultured under HG conditions exhibited a 2.17 ± 0.7-fold increase in ROS production at day 21 compared to NG cells (*p* < 0.0001). This was associated with a 3.79 ± 0.15-fold increase in lipid peroxidation (*p* = 0.0014 vs. NG; Figure 5b).

Interestingly, ROS production remained elevated in MGC cultured under HG-NG conditions (1.54 ± 0.47-fold relative to NG; *p* = 0.0016) despite the return to NG. However, lipid peroxidation in the MGC cultured under HG-NG condition did not differ from cells cultured under NG (*p* = 0.4536). Notably, ROS levels in MGC cultured under HG-NG conditions were lower than in cells cultured under HG (*p* = 0.0006), and lipid peroxidation levels were also reduced compared to cells under HG (*p* = 0.0043), indicating partial recovery for lipid peroxidation but persistence of ROS elevation.

### 3.4. HG Produces an Irreversible Decrease in Mitochondrial Activity in MGC

**Figure 6:**
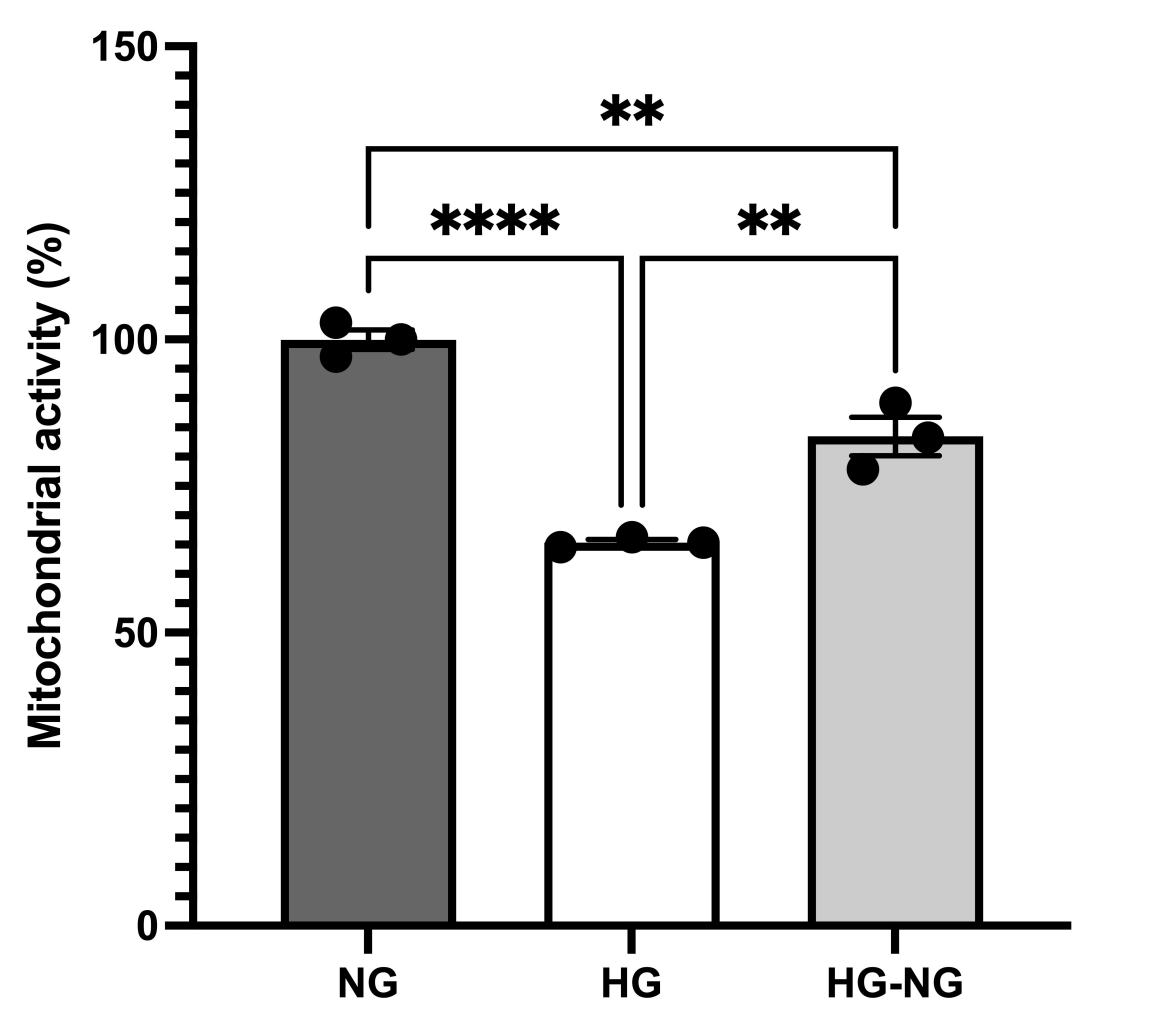
Mitochondrial activity of MGC cultured under different glucose conditions was evaluated using the MTT assay. Data are from three separate experiments and represent the mean ± SEM. Statistical analyses were performed by one-way ANOVA; ** *p* < 0.01 and **** *p* < 0.0001. For between-group comparisons, Tukey’s test was used as a post hoc test.

As shown in Figure 6, MGC cultured under HG and HG-NG conditions exhibited decreases in mitochondrial activity of 36.61 ± 0.48% (*p* < 0.0001) and 16.54 ± 3.27% (*p* = 0.0038), respectively, compared to NG cells. Notably, the reduction in mitochondrial activity in the HG-NG group was less pronounced than in HG cells (*p* = 0.0024), indicating partial recovery upon restoration of NG.

### 3.5. HG Produces an Irreversible Increase in TNF-α Levels

**Figure 7:**
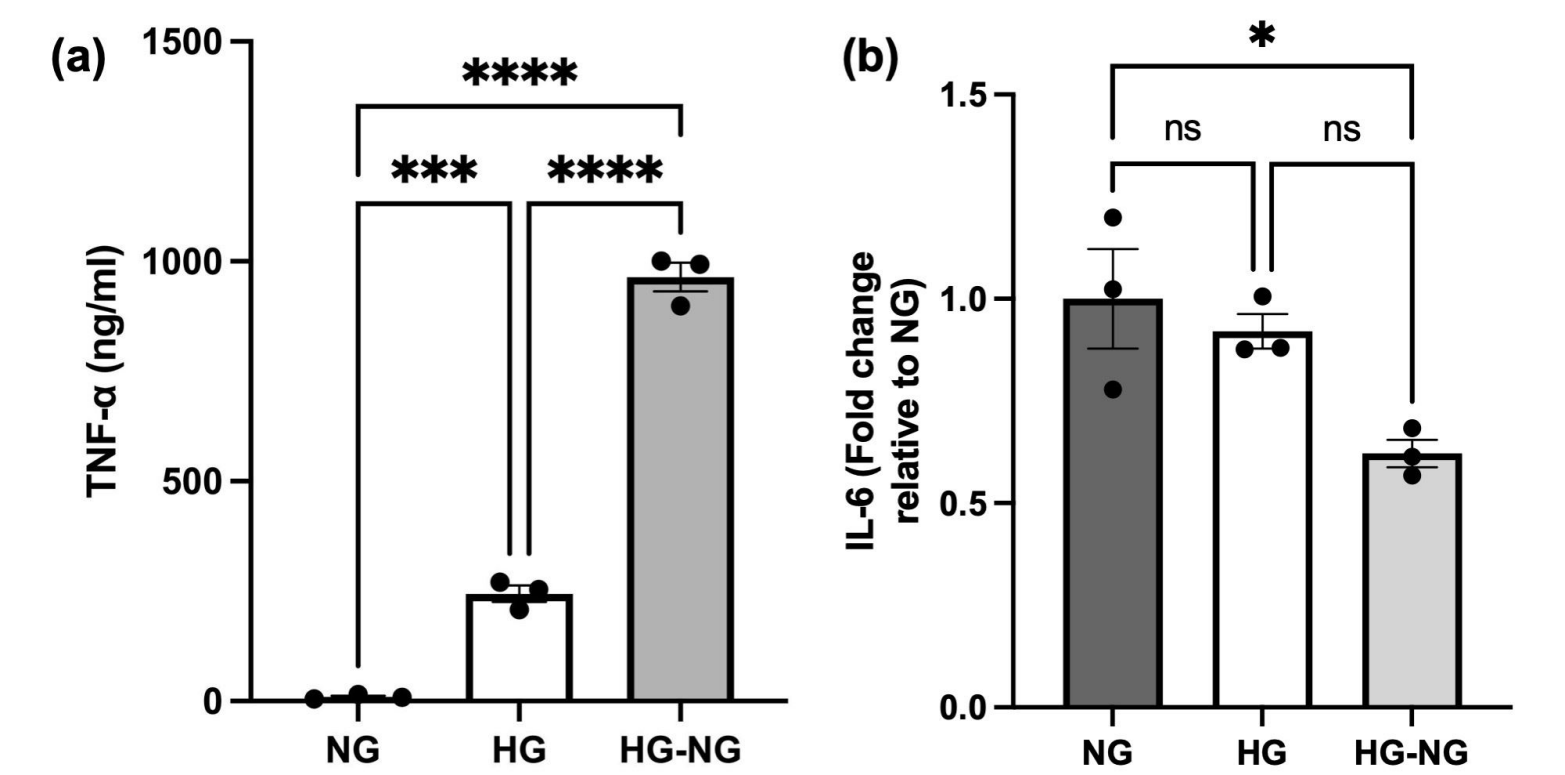
Cytokine production in MGC cultured under different glucose conditions: (**a**) TNF-α levels; (**b**) IL-6 levels. Data are from three separate experiments and represent the mean ± SEM. Data represent the mean ± SEM. Statistical analyses were performed by one-way ANOVA; * *p* < 0.05, *** *p* < 0.001, and **** *p* < 0.0001. For between-group comparisons, Tukey’s test was used as a post hoc test.

As shown in Figure 7a, MGC cultured under HG conditions exhibited a 24.81 ± 1.88-fold increase in TNF-α production, whereas cells in the HG-NG group showed a 97.95 ± 3.31-fold increase compared to NG (*p* = 0.0006 and *p* < 0.0001, respectively). Notably, TNF-α levels were significantly higher in HG-NG cells than in HG cells (*p* < 0.0001).

In contrast, IL-6 production (Figure 7b) did not differ significantly between HG and NG cells (*p* = 0.7566). Interestingly, MGC in the HG-NG group exhibited a significant decrease in IL-6 levels to 0.37 ± 0.03-fold relative to NG (*p* = 0.0307). No significant differences were observed between HG and NG groups for IL-6 (*p* = 0.0750).

### 3.6. HG and HG-NG Favors the AGE Formation

**Figure 8:**
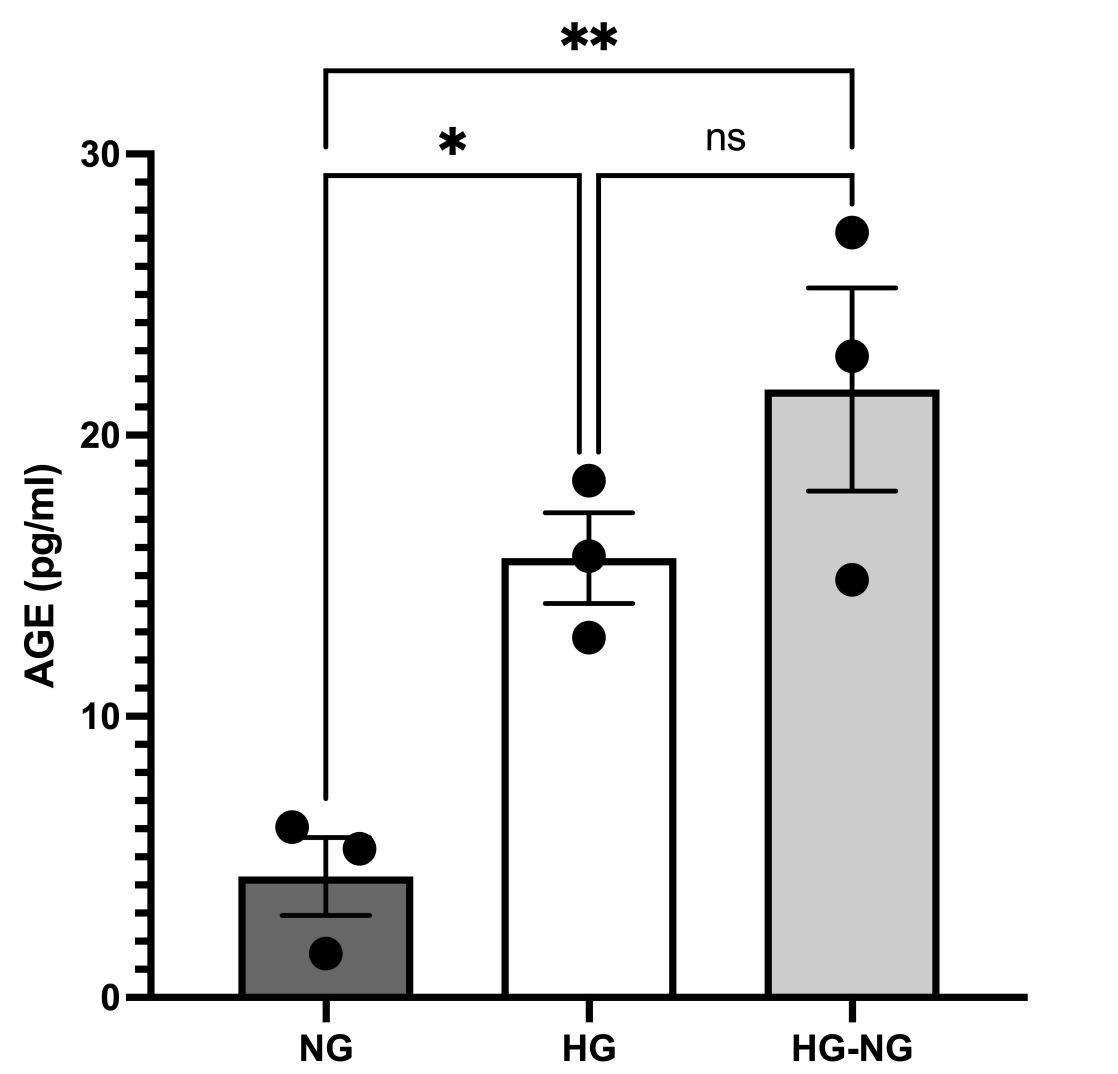
AGE production in MGC cultured under different glucose conditions. Statistical analyses were performed by one-way ANOVA; * *p* < 0.05 and ** *p* < 0.01. For between-group comparisons, Tukey’s test was used as a post hoc test.

As shown in Figure 8, MGC cultured under HG and HG-NG conditions exhibited significant increases in AGE formation, with 3.67 ± 0.37-fold and 5.02 ± 0.83-fold changes relative to NG cells (*p* = 0.0374 and *p* = 0.0056, respectively). No significant difference was observed between the HG and HG-NG groups (*p* = 0.2633).

## 4. Discussion

Recently, there has been growing interest in understanding the effects of diabetes on the brain. Evidence indicates that diabetes negatively affects cognitive function, particularly in individuals diagnosed during childhood [27]. Case–control studies in adults (20–60 years) with type 1 diabetes have reported reductions in brain volume of up to 8% compared with controls [28,29]. Decreases in gray matter density were associated with poor glycemic control, age of onset, and diabetes duration [30], suggesting that cellular-level brain alterations induced by diabetes contribute to the onset and progression of neurodegenerative diseases, including PD, AD, Huntington’s disease (HD), amyotrophic lateral sclerosis (ALS), and other neurodegenerative disorders [31].

In diabetes, all cell types—not only endothelial cells—are exposed to elevated glucose levels. Glucose uptake in several cells, including those in the CNS, occurs via non-insulin-dependent glucose transporters (GLUTs) [32]. The deleterious effects of HG on glial cells have been widely described, including reduced proliferation and increased production of ROS and pro-inflammatory cytokines [11,33].

However, whether these effects persist after restoration of NG levels in glial cells remains unclear—a phenomenon known as “metabolic memory” [34]. Engerman et al. (1987) first described metabolic memory, showing that the incidence of diabetic retinopathy remained high in diabetic dogs even after 2.5 years of normalized blood glucose [12]. Since this initial observation, extensive evidence has corroborated the metabolic memory phenomenon, primarily in endothelial and smooth muscle vascular cells [15,20].

Emerging evidence suggests that other cell types, including astrocytes, may also exhibit metabolic memory. Li W et al. (2018) demonstrated the irreversibility of decreased proliferation in primary astrocyte cultures exposed to HG (25 mM) for 2 days, even after switching to NG (5.5 mM) [7]. Consistent with these findings, the present study demonstrated that HG irreversibly inhibited proliferation in MGC cultures, without affecting apoptosis, confirming that glial proliferation is persistently impaired under hyperglycemic conditions.

A distinctive hallmark of metabolic memory in endothelial cells is the development of oxidative stress. In a study by Ihnat et al. (2007), human umbilical endothelial cells and retinal cells exposed to HG conditions for 2 weeks and subsequently returned to NG for 1 week continued to express markers of HG-induced oxidative stress [15]. Consistently, the present study demonstrated that MGC cultured under HG and HG-NG conditions exhibited increased ROS production, even after glucose normalization during the last 7 days in the HG-NG group. Notably, this increase in ROS correlated with decreased mitochondrial activity in both HG and HG-NG cells, highlighting the role of impaired electron transport under chronic hyperglycemia, which leads to elevated ROS and mitochondrial injury [35].

ROS production in glial cells under HG conditions can also induce lipid peroxidation, damaging membrane lipids [36]. Interestingly, MGC in the HG-NG group did not show increased lipid peroxidation compared to HG cells. This may reflect compensatory mechanisms or the duration of HG exposure. Prolonged HG exposure can trigger antioxidant enzyme responses that mitigate cellular damage [37]. Accordingly, restoration of normal glucose after 2 months of poor glycemic control in streptozotocin-induced diabetic Wistar rats reduced retinal lipid peroxides by ~50%, whereas restoration after 6 months failed to reverse oxidative damage [38]. These findings suggest that the duration of HG exposure is a crucial factor in the development of metabolic memory.

Hyperglycemia also elevates TNF-α production in glial cells, particularly microglia. Quan et al. (2011) showed that primary rat microglia exposed to HG (35 mM) increased TNF-α and monocyte chemotactic protein-1 (MCP-1) secretion via nuclear factor κB (NF-κB) activation, without affecting IL-1β or IL-6 levels [39]. Elevated TNF-α contributes to neuroinflammation and may exacerbate neurodegenerative processes. Similarly, Yao et al. (2022) demonstrated that hyperglycemia in streptozotocin-induced diabetic mice enhances NF-κB signaling in endothelial cells, which persists despite glucose normalization, resulting in perivascular inflammation, fibrosis, and cardiac dysfunction [40]. In the present study, MGC cultured under HG conditions exhibited increased TNF-α production, which persisted after restoration to NG in the HG-NG group, underscoring the irreversibility of pro-inflammatory signaling under metabolic memory conditions.

In contrast, consistent with previous observations in microglia [39], MGC cultured under HG conditions in the present study did not exhibit changes in IL-6 levels compared to NG cells. Interestingly, IL-6 was reduced in MGC cultured under the HG-NG condition. This decrease may reflect deleterious effects induced by prior HG exposure, as low IL-6 levels in the CNS can alter glial activation and impair neuroprotection, particularly during injury or inflammation [41]. This finding warrants further investigation in future studies.

AGEs are a diverse group of glycosylated adducts that can damage cells by cross-linking or modifying intracellular molecules, leading to the accumulation of misfolded proteins [42], and by binding to mitochondrial respiratory chain complexes I and IV, causing mitochondrial dysfunction. Notably, AGE formation has been associated with the establishment of metabolic memory [43]. Ren et al. (2017) demonstrated that AGEs significantly reduce endothelial nitric oxide synthase (eNOS) activity, increase superoxide anion production, decrease mitochondrial membrane potential, and impair antioxidant enzyme activities (catalase and superoxide dismutase), while enhancing nicotinamide adenine dinucleotide phosphate (NADPH) oxidase activity [44]. Consistent with these findings, the present study demonstrated that AGEs are formed in MGC under HG conditions, and this effect persists even after glucose normalization (HG-NG), highlighting the contribution of metabolic memory in glial cells.

## 5. Conclusions

In the present study, we demonstrated that decreased cell proliferation, increased ROS production, impaired mitochondrial activity, and elevated TNF-α production induced by HG exposure were not reversed in MGC cultures. These persistent alterations may contribute to the onset and progression of neurodegenerative diseases.

Finally, the authors acknowledge several limitations of this study, including the small sample size and the lack of assays to assess specific changes among different glial cell types and potential epigenetic modifications. Future studies are warranted to address these gaps and further elucidate the mechanisms underlying metabolic memory in glial cells.

## Data Availability

The raw data supporting the conclusions of this article will be made available by the authors on request.

## Abbreviations

The following abbreviations are used in this manuscript:

AD AGEs	Alzheimer’s disease Advanced Glycation End Products
ANOVA	Analysis of variance
CBA	Cytometric Bead Array
CNS	Central Nervous System
DCFH-DA ELISA eNOS	2′,7′-dichlorofluorescin diacetate Enzyme-Linked immunosorbent assay Endothelial nitric oxide synthase
FBS GFAP	Fetal bovine serum Glial fibrillary acidic protein
GLUTs	Glucose transporters
HG	High Glucose
HG-NG Iba1 MCP-1 MDA	Metabolic memory group Ionized calcium binding adaptor molecule 1 Monocyte chemotactic protein-1 Malondialdehyde
MGC	Mixed Glial cells
MTT NADPH oxidase NF-κB	3-(4,5-dimethylthiazol-2-yl)-2,5-diphenyltetrazol Nicotinamide adenine dinucleotide phosphate oxidase Nuclear factor κB
NG OD	Normal glucose Optical density
PD RIPA ROS	Parkinson’s disease Radioimmunoprecipitation Reactive oxygen species
SOD	Superoxide dismutase
SRB	Sulforhodamine B
TCA	Trichloroacetic acid
TNF-α	Tumor necrosis factor-α
